# OPA1-related dominant optic atrophy is not strongly influenced by mitochondrial DNA background

**DOI:** 10.1186/1471-2350-10-70

**Published:** 2009-07-20

**Authors:** Denis Pierron, Marc Ferré, Christophe Rocher, Arnaud Chevrollier, Pascal Murail, Didier Thoraval, Patrizia Amati-Bonneau, Pascal Reynier, Thierry Letellier

**Affiliations:** 1 Université Bordeaux 1, Laboratoire d'Anthropologie des Populations du Passé, UMR 5199 PACEA, 33400 Talence, France; 2 Institut de Biochimie et Génétique Cellulaires, UMR 5095, CNRS, Université Victor Segalen-Bordeaux 2, 33076 Bordeaux, France; 3 INSERM, U688 Laboratoire de Physiopathologie Mitochondriale; Université Victor Segalen-Bordeaux 2, 33076 Bordeaux, France; 4 INSERM, U694, Angers, F-49933 France; Université d'Angers, Angers, F-49035 France; Département de Biochimie et Génétique, Centre Hospitalier Universitaire, Angers, F-49933 France

## Abstract

**Background:**

Leber's hereditary optic neuropathy (LHON) and autosomal dominant optic atrophy (ADOA) are the most frequent forms of hereditary optic neuropathies. LHON is associated with mitochondrial DNA (mtDNA) mutations whereas ADOA is mainly due to mutations in the OPA1 gene that encodes a mitochondrial protein involved in the mitochondrial inner membrane remodeling. A striking influence of mtDNA haplogroup J on LHON expression has been demonstrated and it has been recently suggested that this haplogroup could also influence ADOA expression. In this study, we have tested the influence of mtDNA backgrounds on OPA1 mutations.

**Methods:**

To define the relationships between OPA1 mutations and mtDNA backgrounds, we determined the haplogroup affiliation of 41 French patients affected by OPA1-related ADOA by control-region sequencing and RFLP survey of their mtDNAs.

**Results:**

The comparison between patient and reference populations did not revealed any significant difference.

**Conclusion:**

Our results argue against a strong influence of mtDNA background on ADOA expression. These data allow to conclude that OPA1 could be considered as a "severe mutation", directly responsible of the optic atrophy, whereas OPA1-negative ADOA and LHON mutations need an external factor(s) to express the pathology (i.e. synergistic interaction with mitochondrial background).

## Background

Hereditary optic atrophies are frequently related to mitochondrial dysfunction. The maternally-transmitted Leber's hereditary optic neuropathy (MIM535000) is associated with one the three main primary point mutations of the mitochondrial genome. With incomplete penetrance, theses mutations induce, between 15 and 35 years old, an acute and bilateral loss of visual acuity with a caecocentral scotoma and a dyschromatopsy. A "sister disease" of LHON, autosomal dominant optic atrophy (MIM #165500) is the second most common form of inherited optic neuropathy, with a frequency of 1:12 000 to 1:50 000 [1,2] This disease is characterized by an insidious onset of bilateral visual impairment in early childhood with moderate to severe loss of visual acuity, temporal optic disc pallor, abnormalities of color vision, and caecocentral visual field scotoma. Electrophysiological and histopathological studies have suggested that the underlying defect is the retinal ganglion cell (RGC) degeneration leading to atrophy of the optic nerve. Until now, four loci have been designated for ADOA [2]. Among these, the most commonly mutated gene is OPA1 (3q28–29) [3,4]. One hundred and seventeen OPA1 gene mutations, mainly family-specific [5], were described with substitutions, deletions and insertions respectively spread throughout the coding sequence of the gene. Most are localized in the GTPase domain and the N-terminus of the protein, whereas the C-terminus is largely spared.

The mitochondrial genetic background is known to influence the expression of LHON [6] since mitochondrial primary mutations G11778A and T14484C showed significant clustering on Caucasian mtDNA haplogroup J. Haplogroup J was also found to be three-fold over-represented in patients with ADOA not related to OPA1 mutations [7].

In order to test the influence of the mitochondrial genetic background on the penetrance of the mutations of the OPA1 gene, we compared the distribution of mtDNA haplogroups between 41 French patients and 1385 individuals of the French population described by Richard and al. [8].

## Methods

### Patients

Screening for ADOA French patients carrying OPA1 mutations was done by the Department of Biochemistry and genetics in the University Hospital of Angers. Genetic analyses were carried out with the appropriate consent of the patients and in compliance with the Helsinki Declaration. Experimental research have been performed with the ethics approval of the " centre hospitalier universitaire d'Angers" and ethics policy of the French mitochondrial disease network [9]. This laboratory collect samples from all French patients suspected to be affected by ADOA. All the patients presented typical history of ADOA with insidious, painless and progressive bilateral visual loss, impairment of visual acuity, central scotoma and optic nerve pallor. OPA1 analysis was carried out by sequencing with the appropriate consent of the patients. Among the cohort of individuals diagnosed as carriers of the OPA1 mutations, we have listed 41 distinct maternal lineages.

### Analysis of the haplogroups

Each patient's DNA was extracted from blood using standard procedures (phenol/chloroform or Qiagene^® ^QIAAmp DNA kit). For each patient, the mitochondrial DNA control region was then amplified using the following primers: L15832 (light chain, nps 15838–15858) and H408 (heavy chain nps 408–429). The results of the amplification were then purified using ExoSAP-IT^® ^technology. Finally, starting from position 16050, at least 725 pb of the mitochondrial DNA control region were double-strand sequenced using an ABI Prism BigDye Terminator Cycle Sequencing Ready Reaction Kit (Perkin Elmer^®^). The same primers were used for amplification and sequencing, L15832 and H408, together with 2 additional primers: L16200 (light chain, nps 16194–16217) and H263 (heavy chain nps 263–285). Each individual was also tested for the presence of a polymorphism in position 7028 by digestion with the AluI enzyme of an amplicon obtained with primers L6909 (light chain, nps 6890–6909) and H7115 (heavy chain nps 7115–7131).

The analysis made it possible to identify each individual's haplotype and haplogroup markers. The individuals' haplogroup affiliation were determined on the basis of recent studies involving total sequencing of mitochondrial DNA [10-13]. Affiliations to the main European haplogroups were confirmed by standard RFLP tests (UK:12308HinfI;JT 4216NlaIII; J: 13704Bsto1; T: 15606AluI; H7025AluI V 4577NlaIII) on diagnostic positions for the haplogroups on the mtDNA coding segment [14,15].

### Control Population and Statistics

The distribution of carriers of mutation on OPA1 gene among the haplogroups was compared with frequencies observed in a sample of the French population consisting of 1385 individuals [8]. Each haplogroup was tested as a possible risk factor for developing the pathology against the rest of the population. In turn statistical comparisons were carried out on the largest cluster, using standard "Khi 2" methods without correction; the haplogroup and sub haplogroup were tested by fisher's exact test. The relative risk estimated for each haplogroup was defined by the "odds ratio" method, with a confidence interval calculated by the Miettinen method [16].

## Results

**Figure 1:**
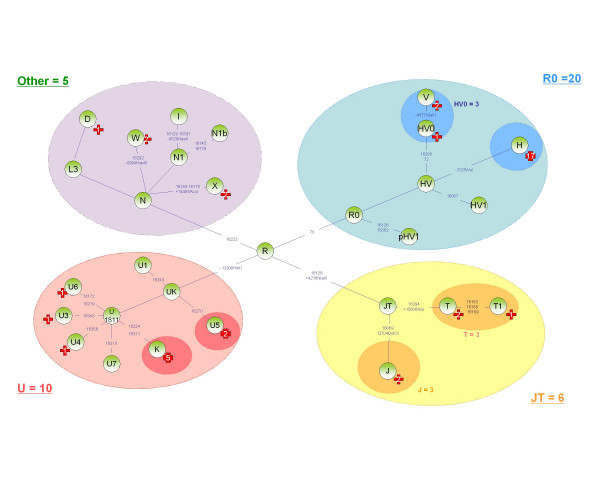
**Phylogenical repartition of the 41 OPA1 mutation carriers**. The phylogenic tree was based on recent total sequencing studies of mitochondrial DNA [11,12]; the new haplogroup nomenclature is used[17]. The different polymorphisms used for haplogroup determination in this study are indicated in blue. The Red Cross indicate the number of individuals per haplogroup.

MtDNA sequencing D-loop and RFLP analysis of the coding segment revealed 37 different haplotypes, among the 41 carriers of a mutation on OPA1 (table 1). The range of haplogroups was extremely wide and included the typically European haplogroups H, preV, V, J, T1, T2, K, U2, U5, U4, X, and W, plus two representatives each of African haplogroup U6, and Asian haplogroup D (figure 1). The presence of the two last haplogroups is expected, since African mtDNAs are common in France and members of Asian super-haplogroup M are sporadically identified in Europe.

**Table 1:** RFLP and Control-Region mtDNA Haplotypes from the French OPA1 mutation carrier

patient	super haplogroup	haplogroup	POLYMORPHISM OBSERVED BETWEEN POSITION 16050–16569 AND 1–205	**RFLP**
1421	R0	H	16519T>C, 195T>C	-7025AluI

1043	R0	H	16519T>C, 146T>C	-7025AluI

1659	R0	H	16519T>C	-7025AluI

1148	R0	H	16519T>C	-7025AluI

1284	R0	H	16311T>C, 16519T>C	-7025AluI

1090	R0	H	16235A>G, 16291C>T, 16293A>G, 16400C>T	-7025AluI

1428	R0	H	16189T>C, 16194-del, 16519T>C	-7025AluI

1568	R0	H	16188C>T, 16295C>T, 16519T>C, 150C>T	-7025AluI

1344	R0	H	16188C>G, 16189T>C, 16264C>T, 16311T>C, 16519T>C	-7025AluI

1267	R0	H	16129G>A, 16264C>T, 16316A>G, 16519T>C, 195T>C	-7025AluI

1366	R0	H	16086T>C, 16189T>C,16519 T>C	-7025AluI

1091	R0	H1a	16162A>G, 16274G>A, 16519T>C, 73A>G	-7025AluI

1163	R0	H1b	16183A>C, 16189T>C, 16291C>T, 16356T>C, 16519T>C, 152T>C	-7025AluI

1531	R0	H5	16184C>T, 16304T>C, 16519T>C	-7025AluI

1686	R0	H5	16243T>C, 16304T>C, 152T>C	-7025AluI

1505	R0	H5	16304T>C, 16519T>C, 146T>C	-7025AluI

1054	R0	H11	16293A>G, 16519T>C	-7025AluI

1032	R0	preV (HV0)	16298T>C, 72T>C	+4577NlaIII, +7025AluI

1620	R0	V (HV0)	16298T>C, 72T>C, 195T>C, 198C>T	+4577NlaIII, +7025AluI

1697	R0	V (HV0)	16298T>C, 16311T>C, 72T>C, 195T>C	-4577NlaIII, +7025AluI

1332	JT	J	16069C>T, 16126T>C, 73A>G, 185G>A	+4216NlaIII; -13704BstOI,+7025AluI

1743	JT	J	16069C>T, 16126T>C, 73A>G, 185G>A	+4216NlaIII; -13704BstOI,+7025AluI

1779	JT	J	16069C>T, 16126T>C, 73A>G, 185G>A	+4216NlaIII; -13704BstOI,+7025AluI

1464	JT	T	16126T>C, 16153G>A, 16294C>T, 16519T>C, 41C>T, 73A>G, 150C>T, 200A>G	+4216NlaIII;+13704BstOI,+7025AluI

1099	JT	T1	16126T>C, 16163A>G, 16186C>T, 16189T>C, 16294C>T, 16519T>C, 73A>G, 152T>C, 195T>C	+4216NlaIII;+13704BstOI,+7025AluI

1763	JT	T2	16126T>C, 16294C>T, 16296C>T, 16304T>C, 16519T>C, 73A>G, 195T>C	+4216NlaIII;+13704BstOI,+7025AluI

1348	U	U3	16343A>G, 16390G>A, 16519T>C, 73A>G, 150C>T	-12308HinfI,+7025AluI

1106	U	U4	16111C>T, 16140T>C, 16356T>C, 16362T>C, 16519T>C, 73A>G, 146T>C, 152T>C, 195T>C	-12308HinfI,+7025AluI

1291	U	U5	16192C>T, 16256C>T, 16270C>T, 16291C>T, 16399A>G, 16519T>C, 73A>G	-12308HinfI,+7025AluI

1742	U	U5 or U4	16270C>T, 16356T>C, 16519T>C, 73A>G, 152T>C, 195T>C	-12308HinfI,+7025AluI

1363	U	U6	16172T>C, 16219A>G, 16261C>T, 16311T>C, 16361G>A, 73A>G	-12308HinfI,+7025AluI

1278	U	K	16093T>C, 16224T>C, 16311T>C, 16519T>C, 73A>G	-12308HinfI,+7025AluI

1455	U	K	16093T>C, 16224T>C, 16311T>C, 16519T>C, 73A>G	-12308HinfI,+7025AluI

1587	U	K	16064T>K, 16129G>A, 16224T>C, 16311T>C, 16519T>C, 73A>G, 180T>Y	-12308HinfI,+7025AluI

1799	U	K	16224T>C, 16311T>C, 16519T>C, 73A>G, 146T>C, 152T>C	-12308HinfI,+7025AluI

1887	U	K	16213G>A, 16224T>C, 16311T>C, 16519T>C, 73A>G, 146T>C	-12308HinfI,+7025AluI

1426	other	D (M)	16260C>T, 16261C>T, 16266C>T, 16301C>T, 16311T>C, 16319G>A, 16362T>C, 16527C>T, 64C>T, 73A>G, 195T>C	+7025AluI

1358	other	W	16083C>T, 16223C>T, 16292C>T, 16519T>C, 73A>G, 106G>A, 189A>G, 195T>C, 204T>C	+7025AluI

1588	other	W	16192C>T, 16223C>T, 16292C>T, 16325T>C, 16519T>C, 73A>G, 189A>G, 194C>T, 195T>C, 204T>C	+7025AluI

1759	other	X	16189T>C, 16194delA, 16223C>T, 16278C>T, 16519T>C, 73A>G, 153A>G, 195T>C	+7025AluI

1512	other	X	16189T>C, 16192C>T, 16223C>T, 16278C>T, 16519T>C, 73A>G, 153A>G, 195T>C	+7025AluI

In light of these results (table 1), we investigated whether there was a difference in distribution among haplogroups of the patient population carrying mutation on OPA1 compared to the reference population. We therefore compared the distribution of 41 mutation carriers among European haplogroups with the frequencies obtained for 1385 unrelated French people [8]. The patients were distributed on a phylogenetic tree of the European population based on recent total sequencing studies of mitochondrial DNA [10-13]. This tree shows the main haplogroups found in the French population. We used the new haplogroup nomenclature recently proposed [17] to place our patients on this tree, indicating the number of individuals per haplogroup (figure 1).

For the purposes of statistical comparison, we collected individuals in phylogenetic clusters centered on super-haplogroup R. This point was chosen as it corresponded to the node where the R line, which represents over 90% of the French population, divides off from the rest of the human phylogeny. We then analyzed whether each cluster constituted a risk factor compared to the rest of the population, using the odds ratio method, with Khi2 to test significance (table 2). We tested the 3 largest clusters representative of a phylogenic reality, so that we could use Khi2 without correction. The statistic analyses of the largest clusters fail to show statistical differences (p > 0.58). To obtain more detailed results, we applied the fisher's exact test to the largest sub-cluster of R0 i.e. haplogroups H and haplogroup HV0. The frequency of haplogroup H and HV0 did not seem significantly different from that observed in the control population (p ≥ 0.44). In view of the lack of data on the proportions of H sub-haplogroups in the French population, as well as the considerable variation in the frequency of sub-haplogroups in Western Europe [11], we have decided not to subdivide this haplogroup. As in the case of haplogroup R0, we tested for significant differences in the sub-cluster of UK and JT haplogroups, however, there was no significant difference for the sub-cluster (p ≥ 0.24).

**Table 2:** Relative risk estimated of each haplogroup to develop the OPA1 pathology against the rest of the population

	OPA1	French	p-value 1	**O.R**	p-value 2	lower 95% CI	Upper 95% CI
	carriers	Population	Fisher		Khi2		
**R0**	**20**	**726**	**0,75**	**0,86**	**0,65**	**0,46**	**1,61**
>H	17	660	0.52				
>HV0	3	66	0.44				
**U**	**10**	**303**	**0.70**	**1,15**	**0,70**	**0,56**	**2,38**
>U5	2	115	0.57				
>K	5	107	0.24				
**JT**	**6**	**224**	**0.89**	**0,89**	**0,79**	**0,37**	**2,14**
>J	3	106	0.95				
>T	3	118	0,86				
**N**	**5**	**132**	**0,58**				

## Discussion

**Figure 2:**
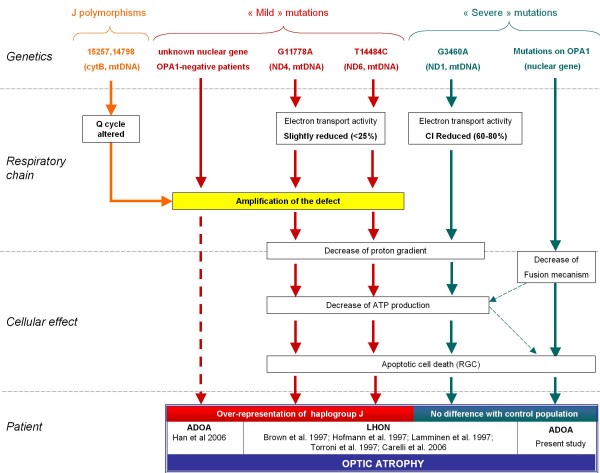
**Influence of mtDNA background on the differentoptic atrophy forms**. The influence of the mtDNA background (i.e., Haplogroup J) may vary according to deleterious effect of a mutation on mitochondrial oxidative phosphorylation. For example, "mild" mutations (i.e., T14484C and G11778A) could have to be amplified by the additional negative effect of mtDNA background (i.e., G15257A, T14798C) to influence the disease expression; where as "severe mutation" (i.e., G3460A, OPA1) does not necessarily need. The dashed spears describe unclear or unproved relations.

The mitochondrial genetic background is known to influence the expression of the optic atrophy: i.e the LHON mitochondrial primary mutations G11778A and T14484C showed significant clustering on Caucasian mtDNA haplogroup J. It has been suggested that this clustering result from an accumulation of non-synonymous J polymorphisms on the cytochrome b gene G15257A, T14798C [18]. Indeed these polymorphisms could affect the complex III efficiency and more specifically the Coenzyme Q binding sites, thus upsetting the proton pump Q cycle and, finally, oxidative phosphorylation (OXPHOS) coupling [19,20]. Consequently J polymorphisms could amplify the effect of the G11778A and T14484C mutations on the biochemical and phenotypical levels (figure 2). A recent study, based on a large cohort of relative patients confirm that the risk of visual failure is greater when the mutations are present in specific subgroups of haplogroup J [21]. However, we could not exclude that the association of the T14484C mutation with haplogroup J might be the result of an elevated specific mutation rate as proposed by [22].

In order to test the influence of the mitochondrial genetic background on the expression of the mutation of the OPA1 gene, we compared the distribution of 41 French patients among European haplogroups with a French population sample constituted of 1385 individuals and described by a recent study [8]. The distribution of the patient population revealed no significant difference compared to the French population.

Interestingly, the clinical expression of the G3460A mtDNA mutation which is also responsible for LHON [23] is not influenced by mtDNA haplogroup J [24-27]. This mutations has a stronger deleterious effect on mitochondrial oxidative phosphorylation (OXPHOS) with a decrease of 60–80% electron transport activity [28]. In contrast, the LHON primary mutations influenced by haplogroup J (T14484C and G11778A) only display a decrease of 0–25% electron transport activity[28]. It is tempting to speculate that the deleterious effect of these later "mild" mutations could have to be amplified by the additional negative effect of mtDNA background to influence the disease expression.

The absence of strong influence of mtDNA background on OPA1-related ADOA expression suggests that, similarly to G3460A mtDNA mutation, the deleterious effect of OPA1 mutations could be responsible itself of the pathology and do not need additional mitochondrial factor. Interestingly, although the main function of OPA1 is devoted to mitochondrial inner membrane remodeling, recent data suggest that mutations of OPA1 could have strong deleterious effect on oxidative phosphorylation efficiency [29,30]. It is tempting to speculate that this energetic defect could be sufficient to induce clinical expression independently to the mtDNA background. Interestingly it has been proposed by Han *et al*. that the expression of OPA1-negative ADOA could remain dependant of additional negative effect of mtDNA background [7]. However, his study was based on an insufficient number of patients and the patients may have a heterogeneous genetic basis, given that multiple loci associated with DOA still await for other genes to be discovered.

## Conclusion

These data allow to conclude that OPA1 should be considered as a "severe mutation", directly responsible of the optic atrophy, whereas OPA1-negative ADOA mutations need an external parameter to express the pathology (i.e. synergistic interaction with mitochondrial background). However, if the influence of mitochondrial background should be excluded for OPA1 mutation, other external parameters as well as environmental or nuclear factors could be implied in the phenotypic expression of the pathology. Although a large study remains to be performed to investigate the possible influences of mtDNA haplogroups on clinical phenotypes of OPA1-related ADOA (ie age of onset, progressivity of the disease, additional neurological symptoms such as neurosensorial deafness), our result indicates the absence of major influence of mtDNA haplogoups in the basic penetrance of the disease. In an attempt to try to better explain the interactions between mitochondrial energetic defect and hereditary optic neuropathies, we propose a model, presented in figure 2, that summarize the influence of mtDNA in optic atrophies.

## Competing interests

The authors declare that they have no competing interests.

## Authors' contributions

DP carried out the molecular genetic studies, statistical analysis and drafted the manuscript. CR, MF, AC participated in the molecular genetic studies. PB, PR participated in the design of the study and drafted the manuscript performed the statistical analysis. PM and DT participated in the design of the study and helped to draft the manuscript. TL conceived of the study, and participated in its design and coordination and helped to draft the manuscript. All authors read and approved the final manuscript

## Pre-publication history

The pre-publication history for this paper can be accessed here:


